# Supplementary material for: An open-source framework for large-scale, flexible evaluation of biomedical text mining systems
Source: J Biomed Discov Collab. 2008 Jan 29;3:1. doi: 10.1186/1747-5333-3-1 (PMC2276192; doi:10.1186/1747-5333-3-1)
Supplement: Additional file 1 — Evaluation platform source code. The additional file contains the software evaluation framework discussed in this paper. The most current release can be downloaded from SourceForge.net [1]. See the accompanying README file for instructions on installation and use. Also included in the distribution is a collection of UIMA wrappers for some commonly used BioNLP tools and annotated corpora. [file 1747-5333-3-1-S1.zip › bionlp_uima_repository-1.0.1/docs/api/edu/uchsc/ccp/uima/cc/summary/AnnotationPrinter.html]

AnnotationPrinter (Center for Computational Pharmacology Util/UIMA API)


|  |  |  |
| --- | --- | --- |
| **Overview** \| **Package** \| **Class** \| **Tree** \| **Deprecated** \| **Index** \| **Help** | |  |
| PREV CLASS   NEXT CLASS | **FRAMES**    **NO FRAMES**     **All Classes** |  |
| SUMMARY: NESTED \| FIELD \| CONSTR \| METHOD | DETAIL: FIELD \| CONSTR \| METHOD |  |


---


## edu.uchsc.ccp.uima.cc.summary Class AnnotationPrinter

```
java.lang.Object
  org.apache.uima.resource.Resource_ImplBase
      org.apache.uima.resource.ConfigurableResource_ImplBase
          org.apache.uima.collection.CasConsumer_ImplBase
              edu.uchsc.ccp.uima.cc.summary.AnnotationPrinter
```

**All Implemented Interfaces:**: org.apache.uima.collection.base\_cpm.CasObjectProcessor, org.apache.uima.collection.base\_cpm.CasProcessor, org.apache.uima.collection.CasConsumer, org.apache.uima.resource.ConfigurableResource, org.apache.uima.resource.Resource

---

``` public class AnnotationPrinter extends org.apache.uima.collection.CasConsumer_ImplBase implements org.apache.uima.collection.base_cpm.CasObjectProcessor ```

This class provides a summary of UIMA processing by outputing the annotations in various formats. Some of these formats are still in development,
and may not work well (for instance, TnT format should be used only when every token has a corresponding annotation), however, the main selections
are:  

- To file or To display- WordFreak Format, TnT Format, or default format

  
The default format should suffice for most uses. The WordFreak and TnT formats were added for the Corpus grant, and have not been fully tested.
  
  

**Author:**
:   William A. Baumgartner, Jr.

---

| **Field Summary** | |
| --- | --- |
| `String` | `PARAM_OUTPUT_INTO_MULTIPLE_FILES` |
| `String` | `PARAM_OUTPUTFILENAME`             The path to the output file must be included in the descriptor under "OutputFileName" |
| `String` | `PARAM_OUTPUTTOFILE`             A boolean flag represented by "OutputToFile" in the descriptor file. |
| `String` | `PARAM_USE_ALT_EVAL_FORMAT` |
| `String` | `PARAM_USE_ANNOTATION_TO_FILE_FORMAT` |
| `String` | `PARAM_USE_BIOCREATIVEFORMAT` |
| `String` | `PARAM_USE_BIOCREATIVENORMFORMAT` |
| `String` | `PARAM_USE_BRILLFORMAT`             A boolean flag represented by "UseTnTFormat" in the descriptor file. |
| `String` | `PARAM_USE_EMBEDDEDXMLFORMAT` |
| `String` | `PARAM_USE_MUTATION_OUTPUT_FORMAT` |
| `String` | `PARAM_USE_ONELINE_FORMAT` |
| `String` | `PARAM_USE_SIMPLE_COLLECTION_READER_FORMAT` |
| `String` | `PARAM_USE_THING_WITH_ENTREZ_ID_FORMAT` |
| `String` | `PARAM_USE_WORDFREAKFORMAT`             A boolean flag represented by "UseWordFreakFormat" in the descriptor file. |

**Fields inherited from interface org.apache.uima.resource.Resource**

`PARAM_AGGREGATE_SOFA_MAPPINGS, PARAM_CONFIG_PARAM_SETTINGS, PARAM_PERFORMANCE_TUNING_SETTINGS, PARAM_RESOURCE_MANAGER, PARAM_UIMA_CONTEXT`


**Constructor Summary**

`AnnotationPrinter()`


| **Method Summary** | |
| --- | --- |
| `void` | `collectionProcessComplete(org.apache.uima.util.ProcessTrace arg0)`             Closes the PrintStream, if one was used by the program. |
| `void` | `initialize()`             Initializes the CAS Consumer by reading in parameters from the descriptor file. |
| `void` | `processCas(org.apache.uima.cas.CAS aCAS)`             Prints out the CAS annotations in the format specified by the descriptor file. |

**Methods inherited from class org.apache.uima.collection.CasConsumer\_ImplBase**

`batchProcessComplete, destroy, getProcessingResourceMetaData, initialize, isReadOnly, isStateless, processCas, reconfigure, typeSystemInit`

**Methods inherited from class org.apache.uima.resource.ConfigurableResource\_ImplBase**

`getConfigParameterValue, getConfigParameterValue, setConfigParameterValue, setConfigParameterValue`

**Methods inherited from class org.apache.uima.resource.Resource\_ImplBase**

`getCasManager, getLogger, getMetaData, getResourceManager, getUimaContext, getUimaContextAdmin, setLogger, setMetaData`

**Methods inherited from class java.lang.Object**

`clone, equals, finalize, getClass, hashCode, notify, notifyAll, toString, wait, wait, wait`

**Methods inherited from interface org.apache.uima.collection.base\_cpm.CasObjectProcessor**

`processCas, typeSystemInit`

**Methods inherited from interface org.apache.uima.collection.base\_cpm.CasProcessor**

`batchProcessComplete, getProcessingResourceMetaData, isReadOnly, isStateless`

**Methods inherited from interface org.apache.uima.resource.ConfigurableResource**

`getConfigParameterValue, getConfigParameterValue, setConfigParameterValue, setConfigParameterValue`

**Methods inherited from interface org.apache.uima.resource.Resource**

`getLogger, getMetaData, getResourceManager, getUimaContext, getUimaContextAdmin, setLogger`

**Field Detail**

### PARAM\_OUTPUTTOFILE

```
public final String PARAM_OUTPUTTOFILE
```

:   A boolean flag represented by "OutputToFile" in the descriptor file. If true, the program outputs to the file specified by "OutputFileName,"
    otherwise, output is directed to the display.

    **See Also:**: Constant Field Values

---


### PARAM\_OUTPUTFILENAME

```
public final String PARAM_OUTPUTFILENAME
```

:   The path to the output file must be included in the descriptor under "OutputFileName"

    **See Also:**: Constant Field Values

---


### PARAM\_USE\_BRILLFORMAT

```
public final String PARAM_USE_BRILLFORMAT
```

:   A boolean flag represented by "UseTnTFormat" in the descriptor file. If true, the program outputs using an embedded format, e.g. token1/tag1
    token2/tag2 ...

    **See Also:**: Constant Field Values

---


### PARAM\_USE\_WORDFREAKFORMAT

```
public final String PARAM_USE_WORDFREAKFORMAT
```

:   A boolean flag represented by "UseWordFreakFormat" in the descriptor file. If true, the program outputs using an XML format similar to
    WordFreak.

    **See Also:**: Constant Field Values

---


### PARAM\_USE\_EMBEDDEDXMLFORMAT

```
public final String PARAM_USE_EMBEDDEDXMLFORMAT
```

**See Also:**: Constant Field Values

---


### PARAM\_USE\_BIOCREATIVEFORMAT

```
public final String PARAM_USE_BIOCREATIVEFORMAT
```

**See Also:**: Constant Field Values

---


### PARAM\_USE\_BIOCREATIVENORMFORMAT

```
public final String PARAM_USE_BIOCREATIVENORMFORMAT
```

**See Also:**: Constant Field Values

---


### PARAM\_OUTPUT\_INTO\_MULTIPLE\_FILES

```
public final String PARAM_OUTPUT_INTO_MULTIPLE_FILES
```

**See Also:**: Constant Field Values

---


### PARAM\_USE\_ONELINE\_FORMAT

```
public final String PARAM_USE_ONELINE_FORMAT
```

**See Also:**: Constant Field Values

---


### PARAM\_USE\_MUTATION\_OUTPUT\_FORMAT

```
public final String PARAM_USE_MUTATION_OUTPUT_FORMAT
```

**See Also:**: Constant Field Values

---


### PARAM\_USE\_THING\_WITH\_ENTREZ\_ID\_FORMAT

```
public final String PARAM_USE_THING_WITH_ENTREZ_ID_FORMAT
```

**See Also:**: Constant Field Values

---


### PARAM\_USE\_ALT\_EVAL\_FORMAT

```
public final String PARAM_USE_ALT_EVAL_FORMAT
```

**See Also:**: Constant Field Values

---


### PARAM\_USE\_SIMPLE\_COLLECTION\_READER\_FORMAT

```
public final String PARAM_USE_SIMPLE_COLLECTION_READER_FORMAT
```

**See Also:**: Constant Field Values

---


### PARAM\_USE\_ANNOTATION\_TO\_FILE\_FORMAT

```
public final String PARAM_USE_ANNOTATION_TO_FILE_FORMAT
```

**See Also:**: Constant Field Values


**Constructor Detail**

### AnnotationPrinter

```
public AnnotationPrinter()
```


**Method Detail**

### initialize

```
public void initialize()
                throws org.apache.uima.resource.ResourceInitializationException
```

:   Initializes the CAS Consumer by reading in parameters from the descriptor file.

    :   **Overrides:**: `initialize` in class `org.apache.uima.collection.CasConsumer_ImplBase`
    :   **Throws:**: `org.apache.uima.resource.ResourceInitializationException`

---


### processCas

```
public void processCas(org.apache.uima.cas.CAS aCAS)
                throws org.apache.uima.resource.ResourceProcessException
```

:   Prints out the CAS annotations in the format specified by the descriptor file.

    :   **Specified by:**: `processCas` in interface `org.apache.uima.collection.base_cpm.CasObjectProcessor`
    :   **Throws:**: `org.apache.uima.resource.ResourceProcessException`

---


### collectionProcessComplete

```
public void collectionProcessComplete(org.apache.uima.util.ProcessTrace arg0)
                               throws org.apache.uima.resource.ResourceProcessException,
                                      IOException
```

:   Closes the PrintStream, if one was used by the program.

    :   **Specified by:**: `collectionProcessComplete` in interface `org.apache.uima.collection.base_cpm.CasProcessor` **Overrides:**: `collectionProcessComplete` in class `org.apache.uima.collection.CasConsumer_ImplBase`
    :   **Throws:**: `org.apache.uima.resource.ResourceProcessException`: `IOException`


---


|  |  |  |
| --- | --- | --- |
| **Overview** \| **Package** \| **Class** \| **Tree** \| **Deprecated** \| **Index** \| **Help** | |  |
| PREV CLASS   NEXT CLASS | **FRAMES**    **NO FRAMES**     **All Classes** |  |
| SUMMARY: NESTED \| FIELD \| CONSTR \| METHOD | DETAIL: FIELD \| CONSTR \| METHOD |  |


---
